# Dielectric Characterization of Healthy Human Teeth from 0.5 to 18 GHz with an Open-Ended Coaxial Probe

**DOI:** 10.3390/s23031617

**Published:** 2023-02-02

**Authors:** Mariya Berezhanska, Daniela M. Godinho, Paulo Maló, Raquel C. Conceição

**Affiliations:** 1 Physics Department, NOVA School of Science and Technology, NOVA University of Lisbon, 2829-516 Caparica, Portugal; 2 Instituto de Biofísica e Engenharia Biomédica, Faculdade de Ciências, Universidade de Lisboa, Campo Grande, 1749-016 Lisbon, Portugal; 3 MALO DENTAL INTERNATIONAL, 1700-029 Lisbon, Portugal

**Keywords:** microwave diagnostics, open ended coaxial probe (OECP), relative permittivity, teeth and dental caries

## Abstract

Dental caries is a major oral health issue which compromises oral health, as it is the main cause of oral pain and tooth loss. Early caries detection is essential for effective clinical intervention. However, methods commonly employed for its diagnosis often fail to detect early caries lesions, which motivates the research for more effective diagnostic solutions. In this work, the relative permittivity of healthy permanent teeth, in caries-prone areas, was studied between 0.5 and 18 GHz. The reliability of such measurements is an important first step to, ultimately, evaluate the feasibility of a microwave device for caries detection. The open-ended coaxial probe technique was employed. Its performance showed to be compromised by the poor probe-tooth contact. We proposed a method based on applying coupling media to reduce this limitation. A decrease in the measured relative permittivity variability was observed when the space between the probe tip and tooth surface was filled by coupling media instead of air. The influence of the experimental conditions in the measurement result was found to be less than 5%. Measurements conducted in ex vivo teeth showed that the relative permittivity of the dental crown and root ranges between 10.0–11.0 and 8.0–9.5, respectively.

## 1. Introduction

Dental caries is a complex disease, characterized by progressive destruction of dental hard tissues due to demineralization of the inorganic component and disintegration of the organic constituents of the tooth by acidic by-products, produced from bacterial fermentation from dietary carbohydrates [1]. This is one of the most prevalent oral diseases worldwide. It is estimated that 2 billion adults and 520 million children suffer from dental caries [2], which compromise quality of life, as it is the main cause of oral pain and tooth loss [3]. It also leads to a high economic burden, as the cost of dental treatment is high due to the required restoration and maintenance throughout life when the tooth structure is destroyed [3]. Thus, early detection and accurate assessment of dental caries are essential for effective clinical intervention, reducing the risk of irreversible loss of tooth structure and, as a result, reducing the cost and treatment time required for restoration [4].

Caries detection is commonly assessed through subjective visual and tactile examination, often supported by X-rays [5]. These methods are effective at detecting caries in advanced states, where tooth restoration is necessary [6], but often show low sensitivity and may fail to detect early caries lesions [5]. In order to detect the initial signs of demineralization, complementary technologies have been developed over the past decades [7,8,9,10,11,12]. These methods assess changes in the optical, electrical or thermal properties of the tooth, due to caries progression, or evaluate the activity of cariogenic bacteria, responsible for tooth demineralization [11]. Although complementary technologies can improve caries detection, they still face some difficulties (such as tooth measurement site, presence of stains or dental plaque) that may limit their performance [13], which motivates research for new and more effective diagnostic solutions, such as Raman spectroscopy and optical coherence tomography [14,15,16].

Over the last decades, microwave sensing and imaging has been investigated as a novel diagnostic technique, as it is a low health-risk method due to the application of nonionizing, low-power electromagnetic signals in the frequency range of hundreds of megahertz to a few gigahertz [17,18]. Microwave imaging has been used in medical applications, including the imaging of breast cancer [19], axillary lymph nodes [20,21,22], brain strokes [23,24] and bones [25]. These medical applications have justified the study of dielectric properties of many biological tissues, including liver [26], breast [27,28] among many others [29,30], and have triggered classification based studies [31,32,33,34,35,36]. Experimental work carried out in the field of dentistry has shown that frequencies in the microwave/millimetre-wave range are able to discriminate between healthy teeth and teeth with visible caries [37,38], which motivates further investigation of microwave diagnostics applicability in detecting healthy teeth and teeth affected by caries. In particular, detailed knowledge of the dielectric properties (relative permittivity and conductivity) of healthy teeth and teeth affected by caries is required, as microwave diagnosis exploits dielectric contrast between tissues to detect pathological conditions [18].

Among currently available methods for dielectric properties measurements of biological tissues, the open-ended coaxial probe (OECP) technique is the most commonly used method due to its simplicity and possibility to perform both ex vivo and in vivo measurements over a broad frequency range [39]. However, the performance of OECP may be limited by some technique’s assumptions, such as perfect contact between the probe and the material under test (MUT) [40]. With an imperfect contact, presence of air gaps within the sensing volume of the probe may induce data inconsistencies when repeated measurements are performed and may cause substantial distortion within measurements [39,40,41] because the dielectric properties of air are low compared to most biological tissues [41]. This may hinder characterization of samples such as teeth, which have an uneven surface, meaning that the sensing volume measures both teeth and air. Nevertheless, OECP was employed by Hoshi et al. [37] to investigate dielectric properties of enamel, dentin and permanent teeth with different stages of dental caries, ex vivo, over the frequency range from 0.04 to 40 GHz. This study [37] found that teeth components are characterized by different dielectric properties, which also differ from tissues at different stages of caries progression; thus, this frequency range could be employed for caries detection. However, caries of different stages of progression were approximated by measuring caries-affected tissue with different degrees of hydration, i.e., by measuring samples at different instants after their removal from the preservative solution. The type of preservative solution used in the study and its possible contribution to the measured dielectric properties have not been reported. Studies conducted by Meaney et al. [41] and Li et al. [42] also applied the OECP technique to characterize ex vivo teeth, but for different dental purposes. For the purpose of biodosimetry, Meaney et al. [41] compared the relative permittivity of the enamel of five incisor and dental resin samples in the frequency range from 0.1 to 8.5 GHz. In this study, the quality of probe contact with the tooth samples was also addressed, as a large variability was noted within each measurement group. The authors reported that given the irregular tooth surface, the measurement results contained inevitable air contributions, which was responsible for adding variability to the measurements. Finally, Li et al. [42] applied OECP with a small aperture for the detection of cracked teeth between 1 and 20 GHz. Although measurements of dielectric properties conducted at multiple locations on the permanent molar crown showed consistency, the authors also assumed the presence of small air gaps between the probe tip and the tooth surface.

The small number of experimental studies carried out in the field of dentistry and their limitations motivate further characterization of human teeth dielectric properties. The knowledge about these properties and the level of dielectric contrast between healthy and carious teeth will allow for the feasible evaluation of microwave-sensing technology for early caries detection. Our study focuses on the measurement of relative permittivity of healthy permanent human teeth with OECP technique. As the performance of the probe may be limited by the quality of contact produced between the probe tip and the tooth surface, OECP suitability for dielectric measurement of teeth was evaluated through a measurement repeatability test. A method based on the application of thin layers of coupling media between the probe tip and the tooth surface was proposed to reduce errors induced by poor probe–sample contact. The proposed methodology was applied to estimate, ex vivo, relative permittivity of four healthy permanent teeth in caries-prone areas.

In Section 2, the experimental measurement system, materials and procedures applied in each measurement scenario are described. Section 3 provides the measurement results and corresponding analysis. Finally, in Section 4, the conclusion is presented.

## 2. Materials and Methods

This section describes the dielectric measurement system and sample characterization. Then, the repeatability study conducted to evaluate OECP performance for teeth dielectric characterization is detailed, and the methodology to estimate the tooth’s relative permittivity from measurements conducted with coupling medium application is presented. We overview the measurements made to evaluate the performance of the method that uses coupling media and the identification of potential confounders such as the irregularity of teeth, the amount of coupling media,uo05+ and measurement time. In addition, the application of the proposed methodology to measure relative permittivity of healthy permanent teeth is explained.

### 2.1. Dielectric Measurement Set-Up

**Figure 1:**
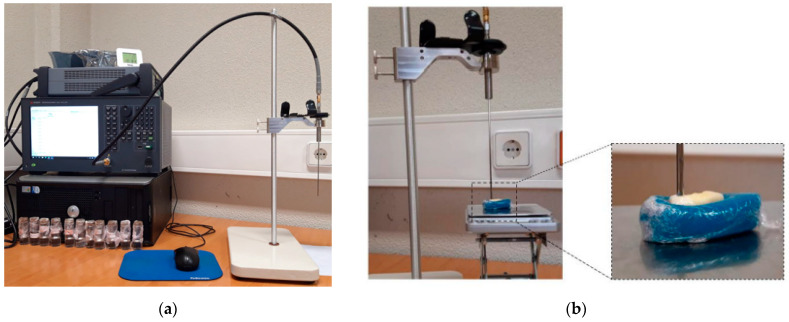
Experimental set-up: (**a**) measurement equipment; (**b**) tooth position during the measurement. Sample is supported by the plasticine holder in order to adjust the measurement site to the probe surface.

The measurement of relative permittivity was conducted using an OECP (Slim Form Probe, N1501A, Keysight, Santa Rosa, CA, USA) connected to a Vector Network Analyzer (VNA) (E5063A, Keysight) through a coaxial cable (Figure 1a). The reflection coefficients (S_11_) measured by the VNA were converted to complex permittivity by Keysight Material Measurement Suite software. During the measurements, 101 linearly spaced points were measured between 500 MHz and 18 GHz, the frequency range supported by the VNA, an IF bandwidth of 30 Hz. To facilitate the positioning of the human teeth under the probe surface during measurements, teeth were placed into plasticine holders coated with cling film, as shown in Figure 1b. The sensing volume of the probe is defined by the radius and the depth extension from the probe tip where materials can be detected by the probe. The sensing volume depends on the properties of the material and the diameter of the probe. The used OECP has a 2.2 mm diameter; the manufacturer recommends samples with 5 mm thickness and 10 mm diameter [40].

The acquisition system was calibrated by the measurement of reflection coefficients: of an open circuit, i.e., the probe surrounded by air; of a short circuit, produced by connecting a short-block to the probe; and of distilled water. The quality of the calibration and its maintenance through the acquisitions was estimated from the comparison between the measured and modelled relative permittivity of the 0.1 M NaCl solution (validation measurements), according to Equation (1):

(1) $$\Delta \varepsilon^{\prime }{\left(f\right)}_{\%}=\frac{\left|{\varepsilon^{\prime }}_{A}\left(f\right)-{\varepsilon^{\prime }}_{B}\left(f\right)\right|}{{\varepsilon^{\prime }}_{A}\left(f\right)}\times 100\;\left(\%\right)$$

where ${\varepsilon^{\prime }}_{A\;}\left(f\right)$ represents the relative permittivity (ɛ′) given by the theoretical model [43], and ${\varepsilon^{\prime }}_{B}\left(f\right)$ represents the relative permittivity acquired during the validation measurement. After calculating the percentage error for each frequency point, $\Delta \varepsilon^{\prime }{\left(f\right)}_{\%}$, the average value of the relative permittivity error was calculated.

### 2.2. Sample Characterization

In our study, healthy permanent teeth without prior restoration or root devitalization were used. All samples were extracted by dentists at Malo Dental as part of patients’ prosthetic treatment, according to the data acquisition protocol established with the clinic. After extraction, the teeth were cleaned with a physiological solution to remove biological fluids and hydrogen peroxide for disinfection. All samples were sealed in sterilized bags, where they remained until measurement. The samples and the measurement sites, characterized in each of the conducted studies, are summarized in Table 1. Studies conducted to evaluate OECP performance for teeth dielectric characterization or the influence of experimental conditions on the measured results were performed on one to two teeth. The dielectric characterization of healthy permanent teeth was performed over four distinct samples, two molars and two premolars, and on root and crown caries-prone areas, in order to verify if tooth type variation influenced its relative permittivity.

All measurements were conducted at room temperature with dried samples. All samples had a thickness and diameter larger than 5 and 7 mm, respectively. Although the sample diameter was below the one recommended by OECP manufacturer (10 mm) [40], some studies [44] suggest that the sensing radius of the used OECP can be only up to 1.5 mm. As a result, considering the small size of our samples, preliminary tests were conducted to confirm that no wave reflection was recorded at the sample edges.

### 2.3. Repeatability of OECP Measurements for Teeth Dielectric Characterization

To evaluate OECP performance for teeth dielectric characterization, a repeatability study was conducted over the crown and the root of two teeth. For each sample, measurements were conducted over the flattest and most regular surfaces of the root and crown in order to maximize the contact between the probe and the tooth. In total, 10 independent measurements were performed for each of the chosen measurement positions. By independent measurement, we mean the repetition of the entire measurement process between two acquisitions, i.e., after a measurement was completed, the probe was detached from the measured point, and the tooth was removed. The probe was cleaned and placed in contact with the desired measurement point in order to perform a new acquisition. All measurements were conducted at room temperature: [20.7 ± 0.2] °C. The validation measurements were performed before and after the completion of each set of 10 acquisitions.

The percentage variation of the relative permittivity over the repeatability test was calculated according to Equation (1), where ${\varepsilon^{\prime }}_{A}\left(f\right)$ represents the upper limit of the recorded relative permittivity range, for a given frequency *f*, and ${\varepsilon^{\prime }}_{B}\left(f\right)\;$represents its lower limit. For the denominator, the highest measured relative permittivity was used, since this acquisition should correspond to the measurement less affected by the dielectric properties of the air. The average value of the relative permittivity variation was also calculated over all frequency points.

### 2.4. Dielectric Measurements with Coupling Medium Application

Given the irregular tooth surface, producing a proper contact between the probe and tooth is challenging, and as a result, the measurements are contaminated with air properties. To overcome this limitation, alternative approaches for dielectric teeth characterization have been investigated. Since taking measurements over artificially smoothed tooth surfaces, produced either by polishing or by cutting, lead to sample modification, we considered using coupling media to reduce errors induced by poor probe-sample contact.

Studies carried out to date regarding the OECP interaction with heterogeneous tissues [44,45] showed that measured dielectric properties range between the dielectric properties of the materials within the sensing volume of the probe. Thus, the introduction of a coupling medium between the probe and MUT will lead to the measurement of the relative permittivity resulting from the contribution of both materials. If the dielectric properties of the coupling medium vary significantly over the measured frequency range, it may allow for the identification of frequency bands where the dielectric properties of two materials are close to each other.

**Figure 2:**
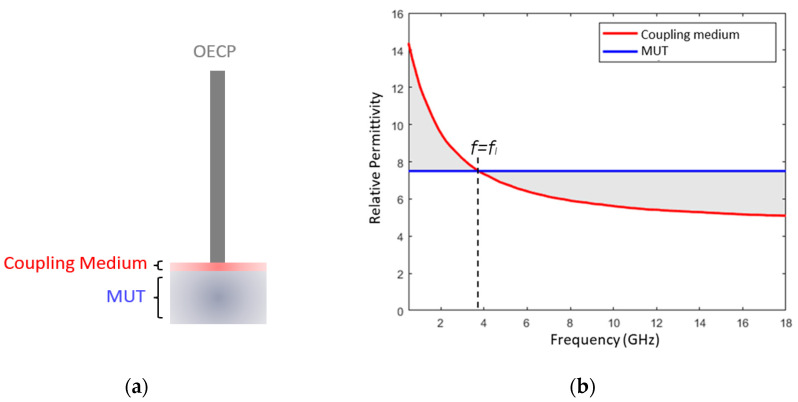
Relative permittivity measurement with coupling medium application: (**a**) schematic representation of the sensing volume of the OECP; (**b**) range of the relative permittivity expected to be measured with coupling medium application over MUT. The sensing volume of the OECP comprises a heterogeneous material formed by two layers: the coupling medium, in direct contact with the probe; and the MUT underneath. The graph illustrates the resulting relative permittivity range expected to be measured (shaded band), where the dielectric properties of heterogeneous material vary between the dielectric properties of the two individual materials present in the sensing volume of the probe. The dielectric properties of the coupling medium and MUT are described by the red and blue curves, respectively, and overlap for the intersection frequency, *f = f_I_*_._

For example, if the relative permittivity of the coupling medium and the MUT are those described by red and blue curves in Figure 2, respectively, it would be expected that the relative permittivity resulting from the application of the coupling medium over the MUT (shaded band in Figure 2) would lie between the range bounded by these curves. Thus, if there is a frequency, *f = f_I_*, for which the relative permittivity of the MUT and the coupling medium are similar, for the frequency range near this point, it is expected that the relative permittivity measured by the probe when coupling medium is applied over MUT (shaded band) is similar to the coupling medium relative permittivity (red line). Thus, for example in Figure 2, if an intersection is observed between the shadowed band and the red line, this suggests the presence of a frequency range where the dielectric properties of the object of interest and the coupling medium are similar. Therefore, it is hypothesized that the use of multiple coupling media with different properties may allow for the identification of dielectric properties that are similar between the coupling medium and the MUT at given frequency ranges. These frequencies could be identified from the intersection points between the curves describing the relative permittivity of the coupling medium and the relative permittivity measured during the application of coupling medium on the MUT. Thus, we hope that the relative permittivity observed for the frequencies at which the intersection points occur, *f = f_I_*, will allow for more reliable estimation of the relative permittivity of MUT such as the tooth, minimizing the error introduced in the measurement by the presence of air gaps.

#### 2.4.1. Characterization of Coupling Media

**Figure 3:**
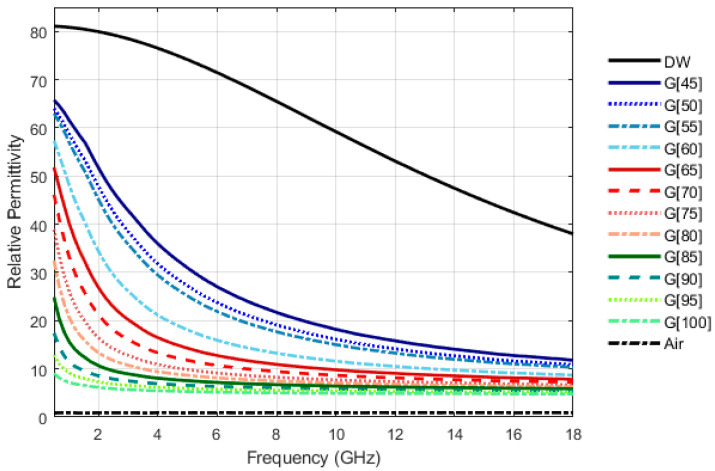
Relative permittivity of produced coupling media at [18.4 ± 0.4] °C. Glycerin volume percentage in the mixtures is given by X in G[X]. Distilled water (DW) and air relative permittivity are presented as a reference.

A total of twelve coupling media have been produced by combining different proportions of vegetable glycerin (Alifar Glycerin) and distilled water (W5 Distilled Water), namely 100:0 (G[100]), 95:5 (G[95]), 90:10 (G[90]), 85:15 (G[85]), 80:20 (G[80]), 75:25 (G[75]), 70:30 (G[70]), 65:35 (G[65]), 60:30 (G[60]), 55:45 (G[55]), 50:50 (G[50]) and 45:55 (G[45]), in percentage by volume. For example, according to the above notation, G[70] corresponds to a solution formed by 70% volume of glycerin and 30% volume of distilled water. The relative permittivity of produced solutions, for the temperature of [18.4 ± 0.4] °C, are shown in Figure 3.

Glycerin is a non-toxic compound [46] that enters in dental pastes composition as humectant and sweetener [47]. Thus, glycerin-based coupling media should not affect teeth properties. Furthermore, it was experimentally confirmed that teeth exposure to glycerin for a period of 24 h does not induce changes in the measured relative permittivity range.

#### 2.4.2. Experimental Protocol and Result Treatment

**Figure 4:**
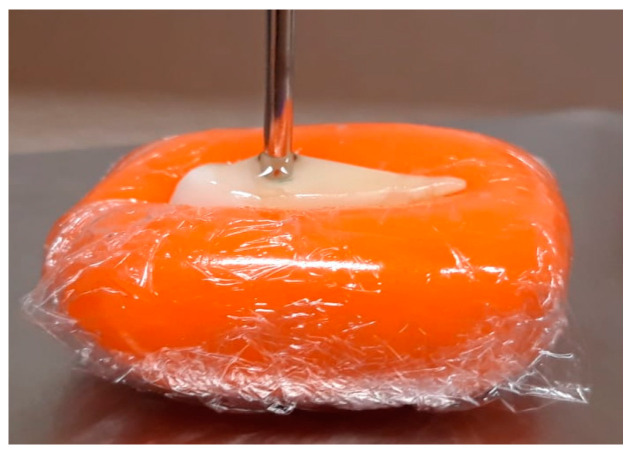
Example of dielectric properties measurement using a coupling medium: a drop of the coupling medium is placed on the tooth surface during the measurement.

To estimate teeth relative permittivity by applying coupling media, for each “coupling medium + tooth” combination, the following protocol is applied:

1. Calibration of measurement equipment.
2. Validation of calibration with 0.1M NaCl solution. Overall, two validation measurements are performed: after equipment calibration and after completion of all measurements.
3. Tooth relative permittivity measurement with application of the coupling medium on the tooth surface. For each combination “coupling medium + tooth” nine measurements were performed according to the following protocol:
  3.1 Air relative permittivity measurement to ensure that the probe was properly cleaned and calibrated.
  3.2 Tooth relative permittivity measurement without coupling medium application. This step allowed for one to obtain a reference of the tooth relative permittivity and to check if the tooth was properly clean, and if it had already been placed in contact with the coupling medium in previous measurements.
  3.3 Tooth relative permittivity measurement with coupling medium application. To this end, the probe is detached from the tooth measurement site, a drop of coupling medium is applied to the tip of the probe, and the dielectric properties are measured (Figure 4). The drop of coupling medium is obtained by immersing the probe into a container filled with the coupling medium and by keeping the drop attached to the probe tip.
  3.4 The probe and the tooth are cleaned with distilled water.
4. To determine the dielectric properties of the coupling medium and monitor its changes, its properties are measured at the beginning, middle and end of the nine acquisitions.

Twenty-three acquisitions were performed for each “coupling medium + tooth” combination: two validation measurements; three measurements of the coupling medium properties; nine measurements of the tooth with the coupling medium; and nine measurements of the tooth without the coupling medium. The measurements of each combination were completed, on average, in two hours. Twelve combinations of coupling medium and tooth were performed for tooth crown dielectric characterization and ten combinations for dental root characterization.

**Figure 5:**
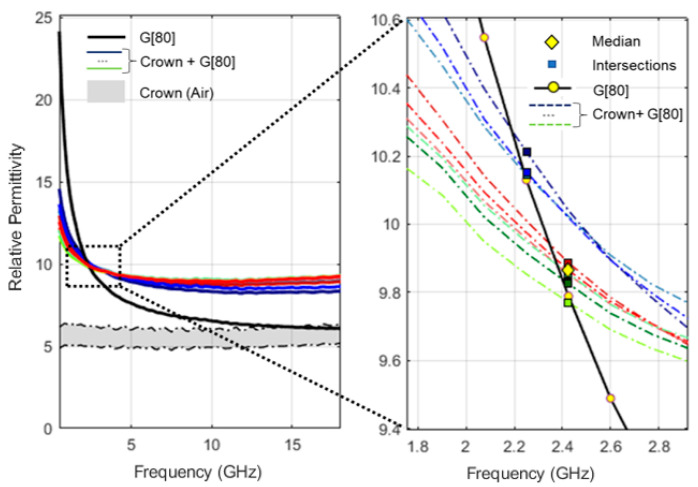
Example of the estimation of the premolar crown relative permittivity with application of coupling medium G[80]. The solid (**left**) and dashed (**right**) lines show the relative permittivity recorded with the application of G[80] over the crown. Relative permittivity of G[80] is represented by a solid black line. The zoomed in region shows intersections (squares) observed for each acquisition. The median value (yellow diamond) of the relative permittivity observed at the intersection points was selected as the estimation of the relative permittivity of the crown. The intersections were determined for the sampled frequencies (yellow circles); thus, there is a slight deviation between the intersections obtained and the points where the interpolated curves intersect. Crown relative permittivity measured without the application of the coupling medium (air) is represented, for reference, by a shaded band.

To estimate the relative permittivity of the teeth, the relative permittivity measured in each of the nine acquisitions for each combination of “coupling medium + tooth” was compared to the average relative permittivity of the tested coupling media. For each acquisition, the intersection point was defined as the frequency for which relative permittivity of the “coupling medium + tooth” was closest to the average relative permittivity of the applied coupling medium. The median value of the relative permittivity recorded at the intersection points was used as an estimation of the relative permittivity of the tooth for the frequency. Figure 5 shows an example of the relative permittivity curves, measured with the application of a coupling medium over the tooth surface, and produced intersections points. It should be noted that for some “coupling medium + tooth” combinations, not all nine measurements intersected with the relative permittivity curve of the applied coupling medium. For these cases, the median value of the relative permittivity was determined only from acquisitions that intersected the relative permittivity curve of the applied coupling medium. If the variability of the relative permittivity observed among all acquisitions, at the frequency point at which the median value was registered, was less than 5% of the median value, this point was considered as the estimation of the tooth’s relative permittivity; otherwise, the intersection was excluded. The exclusion of the intersection point, which happened only twice for higher frequencies (above 16 GHz), only impacts the permittivity estimation for those higher frequencies, not compromising the remaining estimated points.

**Figure 6:**
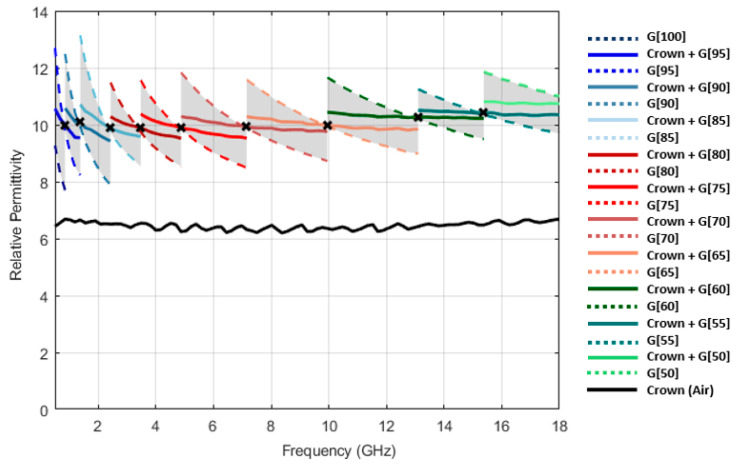
Example of the estimated relative permittivity for a premolar crown with the application of multiple coupling media. For each frequency range delimited by intersection points (black crosses), the relative permittivity of two coupling media (dashed line) and the relative permittivity resulting from the application of these media on the crown (solid line) are shown. The highest measured relative permittivity without application of coupling medium is represented by the solid black line as a reference. Glycerin percentage, by volume, in the coupling medium solution is given by X in the G[X] expression.

The application of multiple coupling media with different properties allowed for the identification of several intersection points (crosses, Figure 6) between the relative permittivity measured with the application of coupling media on the tooth (solid line, Figure 6) and the relative permittivity of the applied coupling medium (dashed line, Figure 6). This allowed for estimating the relative permittivity of the tooth along the frequency range.

### 2.5. Coupling Medium Method’s Performance Evaluation and Potential Confounders Identification

The measurements conducted to evaluate the performance of the proposed method and to identify the potential confounders are detailed in this subsection. Firstly, the methodology applied to test the influence of the irregularity of the tooth surface, and consequently the quality of probe-tooth contact, on the estimated relative permittivity, is presented. Then, we describe the tests conducted to evaluate the influence of some experimental conditions, such as the amount of the coupling medium applied over the tooth surface or measured relative permittivity variation with time elapsed between the application of the coupling medium and the start of the measurement.

#### 2.5.1. Relative Permittivity Variation with the Irregularity of Tooth Surface

**Figure 7:**
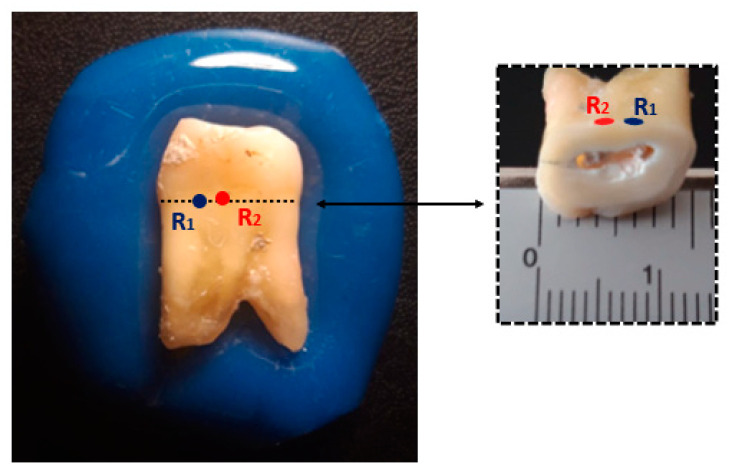
Illustration of the measurement locations on the molar root: R1—smooth and flat surface; R2—concave surface. At the measuring sites, the thickness of the root wall was approximately 2 mm. The pulp cavity had a thickness of approximately 2 mm and was partially filled with devitalized tissue.

To evaluate the dependence of the tooth’s relative permittivity estimation with the quality of OECP contact and, consequently the amount of coupling medium between the probe and the tooth surface, we compared: the estimated relative permittivity over a smooth and flat tooth surface (region with lower medium accumulation—R1 measurement point, Figure 7) and the estimated relative permittivity over a concave tooth surface (region with higher medium accumulation—R2 measurement point, Figure 7). The chosen positions were near each other in order to minimize the tooth structure variation. For the considered sites, measurements were conducted according to the procedure described in Section 2.4.2. Regarding the R1 measurement location, all coupling media from G[100] to G[60] were used. Among these, G[90], G[75] and G[65] were selected to apply on R2. The choice of these coupling media for R2 position was a result of the observation that the relative permittivity measured over R1 produced intersections with the dielectric properties of the applied mixtures, at the beginning (G[90]), middle (G[75]) and end (G[65]) of the considered frequency range. All measurements were conducted at room temperature, [15.3 ± 0.3] °C.

Since the application of equal coupling media for each measurement site (R1 and R2) produced intersections at different frequencies, to quantify the change in relative permittivity recorded between the two measurement sites, the permittivity estimated over R1 was interpolated to the intersection frequencies of R2 for comparison. Thus, the average percentage change of estimated relative permittivity between R2 and R1 sites was computed by averaging the percentage changes obtained for each of the intersection frequencies from the R2 measurement sites. For comparison, percentage variation of the relative permittivity recorded without the application of coupling media (i.e., in the presence of air between OECP and the tooth) was calculated according to Equation (1), where ${\varepsilon^{\prime }}_{A\;}\left(f\right)$ and ${\varepsilon^{\prime }}_{B}\left(f\right)$ were replaced by the highest relative permittivity recorded at the R1 and R2 measurement positions, respectively, without applying any coupling media. The average value of the relative permittivity variation was also calculated over all frequency points. Besides quantifying relative permittivity variation between measurement sites, relative permittivity estimated over R1 was also used to evaluate the variability of the relative permittivity recorded with the application of the coupling media between the different acquisitions over the same measurement site. To this end, the average percentage change in relative permittivity, Δε′(*f*), was calculated between the median measurement, ε′*_M_*(*f*), and the remaining eight measurements, ε′*_i_*(*f*), taken for each “coupling medium + tooth” combination, according to Equation (2):

(2) $$\Delta \varepsilon^{\prime }{\left(f\right)}_{\;}=100\times \frac{1}{8}\overset{8}{\underset{i=1}{\sum }}\frac{\left|{\varepsilon^{\prime }}_{i}\left(f\right)-{\varepsilon^{\prime }}_{M}\left(f\right)\right|}{{\varepsilon^{\prime }}_{M}\left(f\right)}\left(\%\right)$$

#### 2.5.2. Relative Permittivity Variation with the Amount of Coupling Medium and Measurement Time

To study the dependence of the measured relative permittivity given the amount of coupling medium applied to the tooth surface (Test A), we compared: the relative permittivity recorded with the application of the largest amount of the coupling medium feasible to be placed on the tooth surface (a drop attached to the probe tip plus coupling media covering the tooth surface and probe surroundings); the relative permittivity measured by applying the smallest amount of coupling medium (a drop attached to the probe tip), so that it remained only between the probe and the tooth surface. Care was taken not to touch the probe, so that its position was stable. The entire measurement process lasted less than one minute.

To study the variation of the measured relative permittivity with the exposure of the coupling medium to the environment conditions, for instance, temperature changes (Test B), we evaluated the variation of measured relative permittivity with time elapsed between the application of the coupling medium and the start of the measurement. For this purpose, we compared: the relative permittivity measured immediately after the application of the coupling medium on the tooth (reference measurement); the relative permittivity measured one minute and twenty seconds after the reference measurement (delayed measurement). This interval was chosen because it is much longer than the time usually taken between the application of the coupling medium on the tooth and the beginning of the measurement (20/30 s).

Both tests were conducted with six coupling media with different proportions of glycerine and water (G[100], G[90], G[80], G[70], G[60], G[50]). For each combination of “coupling medium + tooth”, the measurement process was repeated three times. All measurements were conducted at room temperature, which was [19.4 ± 0.2] °C for Test A and [19.3 ± 0.5] °C for Test B. The average difference observed between the coupling medium and room temperature at the measurement time was [0.3 ± 0.1] °C. The validation measurements were performed before and after measuring each “coupling medium + tooth” combination.

To estimate the error induced by each of the evaluated conditions, the percentage variation of the relative permittivity was calculated according to Equation (1). In Test A, ${\varepsilon^{\prime }}_{A}\left(f\right)$ and ${\varepsilon^{\prime }}_{B}\left(f\right)$ were replaced by the relative permittivity measured with application of the largest and smallest amount of coupling medium, for a given frequency, *f*, respectively, while for Test B, ${\varepsilon^{\prime }}_{A}\left(f\right)$ and ${\varepsilon^{\prime }}_{B}\left(f\right)$ were replaced by the relative permittivity measured for reference and delayed measurement, respectively. The average value of the relative permittivity variation was also calculated over all frequency points, Δε′(%).

### 2.6. Dielectric Characterization of Healthy Teeth

**Figure 8:**
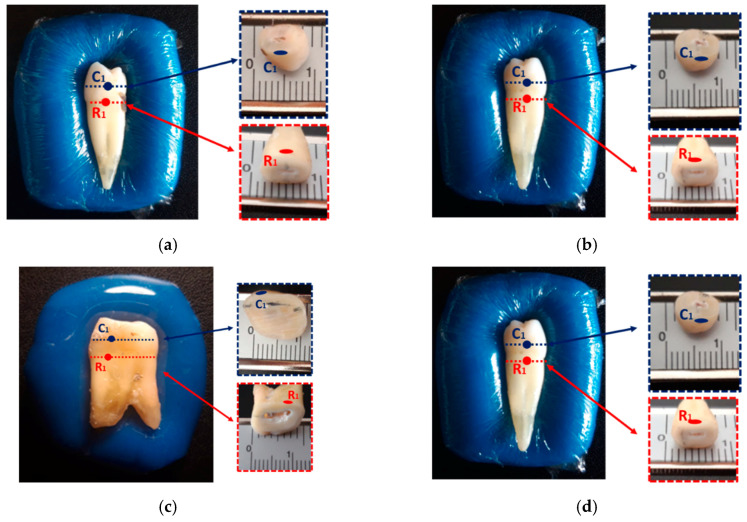
Illustration of the measurement locations on the crown (C1) and root (R1) of dental samples: (**a**) sample PM1; (**b**) sample PM2; (**c**) sample M1; (**d**) sample M2. At measuring sites, the crown and root wall thickness were approximately 2 mm. The pulp cavity of the crown and premolars root had a thickness of approximately 1 mm, while pulp cavity of the molar root measured 2 mm. The pulp cavity was partially filled with devitalized tissue. For the molar crown, the thickness of the pulp cavity was less than 0.5 mm.

The proposed method was applied for the characterization of four healthy teeth, two premolars (PM1 and PM2) and two molars (M1 and M2) at caries-prone sites: one on the crown (C1), between the middle and cervical thirds of the smooth crown surface; and the other on the cervical third of the tooth root (R1). The locations of the measurement points, for each sample, are illustrated in Figure 8.

The selected measurement points were on surfaces as flat and smooth as possible, in order to maximize the probe–tooth contact. In the root, the choice of the cervical third for acquisitions was due to the fact that root caries typically develop on the cervical area, due to exposure of the cementum, after gingival recession [48,49]. In the crown, caries usually develop on smooth surfaces, particularly in the contact points between adjacent teeth, and at the fissures of the occlusal surface [49]. As the choice of the measurement site is limited by the need to maximize contact between the probe and tooth, it was only possible to characterize the smooth surfaces of the crown.

The measurements were conducted according to the procedure described in Section 2.4.2. For the root, all coupling media from G[100] to G[60] were used, while for the crown, combinations from G[100] to G[50] were applied for samples M1, PM1 and PM2, and combinations from G[100] to G[45] were applied for sample M2. The acquisitions were conducted at room temperature, which ranged between 14.3 and 19.5 °C, throughout the acquisition sessions. Although temperature variation was approximately 5 °C, no changes in the range of teeth’s relative permittivity were detected throughout the measurement sessions. However, a variation in the dielectric properties of the coupling medium was observed. Thus, in order to ensure that the dielectric properties were known, the relative permittivity of the coupling media was remeasured several times throughout the measurement sessions. Thus, the observed temperature variations are expected to have no impact on the results.

In order to compare the relative permittivity estimated in the present study with the relative permittivity available in the literature, the relative permittivity percentage change was determined according to Equation (1), where ${\varepsilon^{\prime }}_{A}\left(f\right)$ e ${\varepsilon^{\prime }}_{B}\left(f\right)$ were replaced by the relative permittivity estimated in the present study and the relative permittivity available in the literature [37], respectively. The average value of the relative permittivity variation was also calculated over frequency.

## 3. Results and Discussion

In this section, firstly, the results concerning the OECP performance for teeth dielectric characterization are discussed. Then, the performance of the coupling medium method is presented, and the influence of potential confounders is discussed. Finally, the estimated relative permittivity of healthy permanent teeth is presented.

### 3.1. Repeatability of OECP Measurements for Teeth Dielectric Characterization

**Figure 9:**
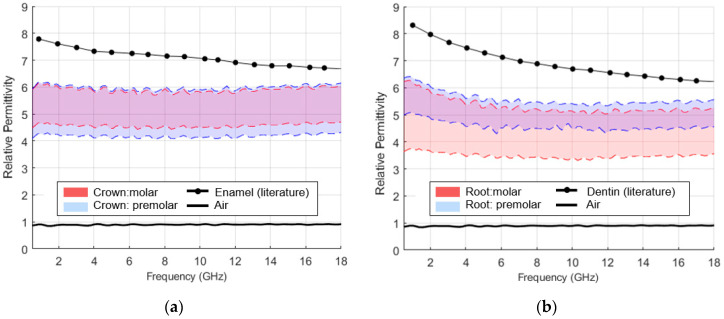
Relative permittivity recorded throughout the repeatability study: (**a**) on the dental crown; (**b**) on the dental root. The measurements performed on the molar and premolar are represented by red and blue shaded bands, respectively. The upper and lower limit of the recorded relative permittivity range, for each of the measurement positions, is shown by dashed lines. The dielectric properties of air, dentin and enamel, available in the literature [37], are presented for reference.

The range of relative permittivity recorded during the repeatability study where the teeth are measured with no coupling media can be found in Figure 9, along the relative permittivity of air, enamel and dentine available in the literature [37].

For the considered frequency range, the recorded relative permittivity average percentage variation ranged from 18.1% to 34.5%, for the considered measurement sites. Furthermore, the highest relative permittivity recorded, for both crown and root, was below the values reported by Hoshi et al. [37] for the frequencies between 0.5 and 18 GHz. The relative permittivity below the value reported in the literature, along with observed variability, suggests poor contact between OECP and tooth surfaces and, as a result, the interference of air in the measurements. The relative permittivity variability was also reported by Meaney et al. [41], whose study suggested the air interference as the main cause of recorded variability in measured relative permittivity. Although an approximately flat surface was chosen for measurements, the tooth surface is quite irregular; thus, it is challenging to have complete contact with the probe. This irregularity may lead to poorer contact and, therefore, to a greater or lesser influence of air on the measurements, which may have been reflected in the measured variability.

This result highlights the difficulty associated with characterizing teeth dielectric properties with OECP. Thus, if the acquisition system is formed only by a probe in direct contact with the tooth sample, the result will be compromised by the presence of air.

### 3.2. Coupling Medium Method’s Performance Evaluation and Potential Confounders Identification

According to the proposed methodology, the use of the relative permittivity observed at the intersection points between the relative permittivity measured with the application of the coupling medium on the tooth and the relative permittivity of the medium, as an estimation of the relative permittivity of the tooth, should reduce the error induced by poor contact between the probe and tooth surfaces. Therefore, the frequency range near the intersection point should lead to a reduction of the variability observed between different acquisitions of tooth relative permittivity. These zones would correspond to frequencies where the dielectric properties of the coupling medium and the tooth are similar.

**Figure 10:**
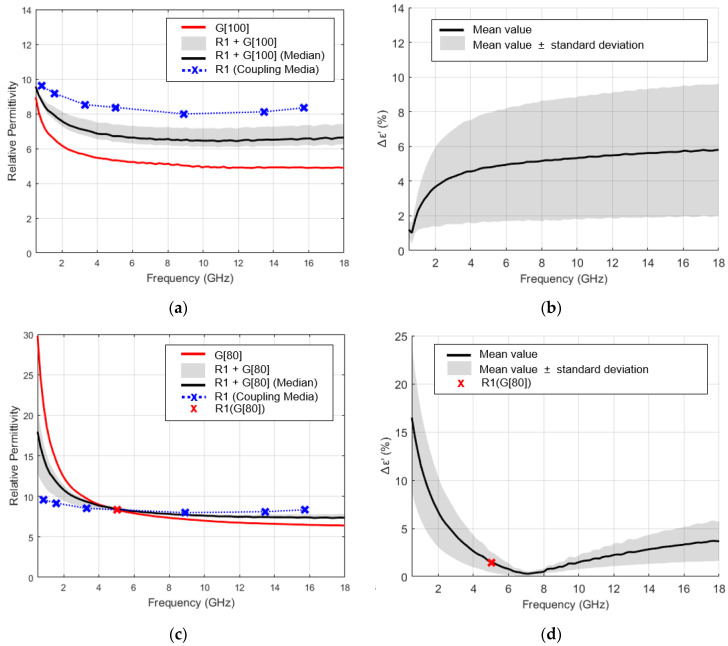
Evaluation of the performance of the method to characterize teeth relative permittivity with the coupling medium. Relative permittivity recorded over molar root (R1 measurement point) with the application of: (**a**) G[100]; (**c**) G[80]. Average percentage variation of the recorded relative permittivity, Δε′ (%), with application of: (**b**) G[100]; (**d**) G[80]. Plots (**a**,**c**) show the median acquisition represented by the black line over the range of relative permittivity measured with the application of the coupling medium (shaded band). The relative permittivity of the exemplified coupling medium is represented by the red line. The relative permittivity estimated for the tooth, according to the proposed methodology, is represented by crosses over blue dashed line. The relative permittivity estimated with the application of the exemplified coupling medium is marked by a red cross, when it occurs. The average percentage variation of the recorded relative permittivity (black line in (**b**,**d**) plots) was taken by the average difference between the relative permittivity recorded for each of the measurements and median measurement. The standard deviation of this variation is shown in the shaded band.

Figure 10a,c shows the example of the relative permittivity range measured with the application of coupling medium, G[100] and G[80], over R1 measurement position (shaded bands), the relative permittivity of the applied coupling medium (red line) and the relative permittivity estimated for the tooth, according to the proposed methodology (crosses over blue dashed line). Figure 10b,d shows the average percentage variation of the measured relative permittivity with the application of the coupling medium, Δε′ (%) (continuous black trace), obtained according to Equation (2). Regarding medium G[100], whose relative permittivity is always below the relative permittivity estimated for the tooth, a reduction in the relative permittivity variability was observed at low frequencies. Regarding medium G[80], whose application leads to an intersection point, a reduction in the relative permittivity variability was observed for frequencies near the intersection (red cross). These results, along with the observation that dielectric properties measured by OECP over a heterogeneous MUT range between the dielectric properties of the materials comprised in the sensing volume of the probe [44,45], suggest an approximation between the relative permittivity of the applied coupling medium and the tooth, for frequencies at which intersection points are observed. However, the intersection did not correspond to the minimum of the relative permittivity variability observed among acquisitions (Figure 10d). This could be associated with the influence of some external factors, such as the error induced by the conditions under which the measurements were taken, or the amount of the coupling medium retained between the probe and the tooth surfaces.

#### 3.2.1. Relative Permittivity Variation with the Irregularity of Tooth Surface

**Figure 11:**
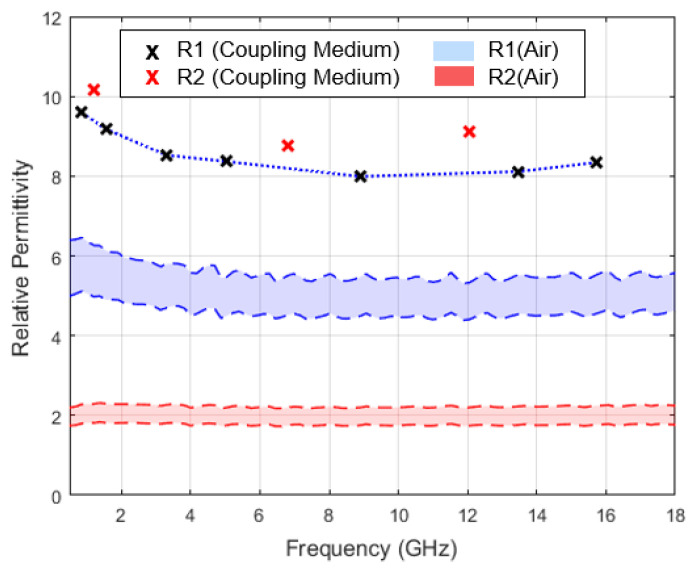
Evaluation of the performance of the method based on coupling media application over two different tooth surfaces. Measurements were conducted over smooth (R1) and concave (R2) surfaces of a molar root. Range of relative permittivity recorded without the application of coupling media (shaded bands) demonstrates a clear difference between tooth–probe contact for the chosen measurement locations. The estimated relative permittivity, with the application of coupling media, is represented by crosses.

Figure 11 shows the relative permittivity estimated for the considered measurement positions. A relative permittivity of approximately 2 was recorded on the R2 measurement point, without the application of coupling media (red band). This suggests a stronger air interference over R2. A poorer contact was observed with probe tip in this position, which led to a greater amount of coupling medium, compared to the R1 measurement position. Higher coupling medium content under the probe led to a systematically higher relative permittivity (blue band). The chosen measurement positions were near each other, and the sample cross-section showed no macroscopic changes of tooth composition (Figure 7). However, an average increase of 9.3% was observed in the estimated relative permittivity with the application of coupling media over R2 in comparison to R1 measurement point. In contrast, without the application of coupling media, this variation was 60.1%.

The result in Figure 11 shows that the amount of coupling medium retained between the probe and the tooth represents a variability factor. However, even for the measurement location that led to excessive accumulation of coupling medium, the variation recorded for the estimated relative permittivity, when compared to the measurement location with better tooth–probe contact, was lower than the variation observed without the application of coupling media. As mentioned before, the dielectric properties measured by the OECP over a heterogeneous MUT range between the dielectric properties of the materials comprised in the sensing volume of the probe [44,45]. Thus, the observation of greater relative permittivity variation in the presence of air (measurements without a coupling medium) between measurement locations with greater (R1) and poorer (R2) probe contact suggests that the air properties differ more from the tooth properties than the coupling media properties observed at the intersection points. Therefore, measurements conducted without the application of coupling media will lead to a distorted relative permittivity in comparison to when coupling media are applied.

#### 3.2.2. Dielectric Properties Variation with the Amount of Coupling Medium and Measurement Time

The average percentage variation of relative permittivity was estimated with: the amount of coupling medium applied on the tooth surface (Test A); and the time elapsed (t = 80 s) between the application of the coupling medium to the tooth and the start of the measurement (Test B). Table 2 shows the variations recorded for the tested combinations of coupling media.

The variation in the measured relative permittivity, caused by either the amount of coupling medium applied over the tooth surface or by the time elapsed between the application of the coupling medium and the beginning of the measurement, was less than 5%, the highest acceptable error established for dielectric properties measurements. Therefore, it is expected that the conditions under which the measurements were taken, in this study, did not significantly interfere with the reported results.

### 3.3. Dielectric Characterization of Healthy Tooth

**Figure 12:**
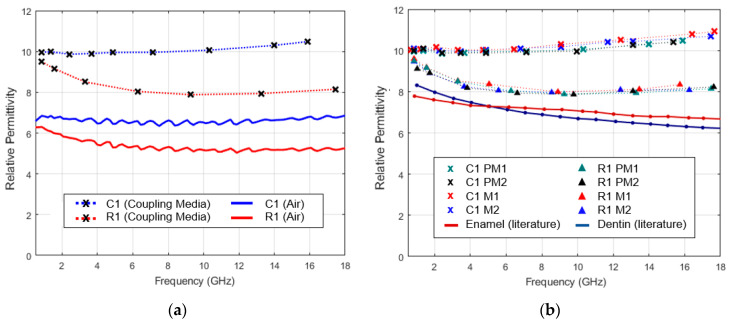
Comparison of the estimated relative permittivity: (**a**) with and without the application of coupling media; (**b**) with the relative permittivity available in the literature [37]. A total of four teeth were measured, two premolars (PMX) and two molars (MX), on the crown (C1, crosses) and root (R1, triangles) caries-prone areas. X denotes the sample identifier.

Figure 12a shows the relative permittivity estimated with the application of coupling media on the crown (C1) and root (R1) of the PM1 premolar sample, along with the maximum relative permittivity recorded for each measurement site without the application of coupling medium (in air). Figure 12b shows the estimated relative permittivity for each sample over caries-prone areas on the crown (C1) and the root (R1). The relative permittivity of enamel and dentin reported in the literature [37] is presented for comparison.

An overlap can be observed between relative permittivity estimated within each of the measurement sets, root (R1) and crown (C1). In addition, a clear distinction is observed between the relative permittivity estimated for the root and the crown, at higher frequencies, which becomes less noticeable for lower frequencies. This pattern is also found in the presence of air (Figure 11a). Regarding root measurement positions (R1), the estimated relative permittivity decreases with frequency from 9.5 to values around 8.0. Regarding measurement sites on the crown (C1), the relative permittivity shows a slight increase, oscillating around 10.0, for lower frequencies, and tending toward 11.0 as the frequency increases. As to the difference between the estimated relative permittivity and dielectric properties available in the literature [37], an average increase of 16.9% for the root and 40.0% for the crown was observed for the considered frequency range. The authors of the study [19] also used OECP for teeth characterization. Given the irregular tooth surface and measurement dependence on the probe–tooth contact quality, an increase in the reported values compared to the literature was expected. One must note that only four teeth were evaluated in our experiments, which limits the generalization of the obtained results. However, these results show the feasibility of our method and motivates its usage in future measurement campaigns.

## 4. Conclusions

This study focused on the measurement of the relative permittivity of healthy permanent human teeth with the OECP technique, whose knowledge is essential to evaluate the feasibility of a caries detection device working in the microwave frequency range. OECP performance in the dielectric measurement of teeth was evaluated, and a method based on the application of coupling media between the probe tip and tooth surface was proposed in order to reduce errors induced by poor probe–sample contact. The experimental protocol was developed, and potential confounders were identified and evaluated. The error introduced by experimental conditions, such as the amount of coupling medium applied over the tooth surface or relative permittivity variation with time elapsed between the application of the coupling medium and the start of the measurement, was found to be comparable to the error introduced by the measurement system, i.e., error below 5%. The quality of contact produced between the probe tip and the tooth surface remained a factor of variability. However, the experimental results showed that the proposed methodology led to a reduction in the variability of measured relative permittivity when the space between the probe and the tooth surface was filled by the coupling medium instead of by air. This outcome highlighted the ability of the developed method to minimize the error associated with poor probe–tooth contact and, therefore, showed the advantage of its employment in the measurement of dielectric properties of irregular solids.

The proposed methodology was applied to characterize the relative permittivity of four healthy permanent teeth, ex vivo and between 0.5 and 18 GHz. Measurements carried out at caries-prone areas showed that the relative permittivity of the dental crown varies between 10.0 and 11.0, while the relative permittivity of dental root is found to be between 9.5 and 8.0, for the considered frequency range. No measurements were performed for teeth with caries, limiting the prediction of the usefulness of this method for caries assessment.

Future work should comprise increasing the sample size and including teeth with different degrees of caries, in order to assess whether there is a dielectric contrast between healthy and carious teeth, and how this contrast evolves with the extent of tooth decay. Carious teeth may present a large variability of characteristics and, consequently, will have different dielectric properties, mainly due to the caries size, its position and depth within each tooth. In addition, deterioration of the teeth may hamper probe–teeth contact, but this type of caries would be visually detected, and an imaging diagnostic tool would not be required. Since the dielectric properties of tissues vary with temperature and degree of hydration, measurements under conditions closer to those found in the oral cavity should be performed.

## Figures and Tables

**Table 1:** Characterization of samples included in each of the studies.

Study	Tooth	Crown	Root
Repeatability of OECP measurements for teeth dielectric characterization	1 Molar and 1 Premolar	Middle to cervical third	Cervical third
Coupling medium method’s performance evaluation and potential confounders identification	1 Molar	-	Cervical third
Dielectric characterization of healthy teeth	2 Molars and 2 Premolars	Middle to cervical third	Cervical third

**Table 2:** Average percentage variation of the relative permittivity, $\Delta \varepsilon^{\prime }\left(\%\right),$, Estimated with: Test A—amount of coupling medium applied on the tooth surface; Test B—time elapsed between the application of the coupling medium on the tooth surface and the beginning of the measurement.

Coupling Medium	$\Delta \varepsilon^{\prime }\left(\%\right),$	
	Test A	Test B
G[100]	1.5	1.2
G[90]	1.4	0.6
G[80]	1.1	0.3
G[70]	1.3	0.2
G[60]	1.0	0.2
G[50]	0.8	0.2

## Data Availability

Not applicable.
